# The Blood

**Published:** 1869-07

**Authors:** Geo. Fisk Keesee


### ARTICLE II.

### *The Blood.*

By GEO. FISK KEESEE, D.D.S.

The human body requires for its maintenance the various processes of digestion, absorption, secretion, excretion, circulation and respiration, which in connection with the several organs and parts of the body fulfil their destined work in the formation, movement, and purification of the blood which is the great motive power of the body.

Blood, the nutritive fluid of the tissues, may be seen circulating in the vessels of a living part as a colorless fluid, but containing small particles, red predominating, which give to the blood a characteristic color. To this colorless fluid has been given the name of liquor sanguinis, and in this fluid is suspended the blood and lymph corpuscles. The liquor sanguinis consists of water in which are dissolved fibrine, albumen, chlorides of sodium and potassium, phosphates of soda, lime and magnesia. Blood when flowing from a wound is a thickish, heavy fluid presenting two different appearances as it may proceed from an artery or a vein, when from the former, it is of a beautiful bright scarlet color, but of a very deep purple when from the latter; these colors, however, are subject to many changes, as the blood may proceed from deep wounds or from asphyxiated persons, but the natural color will be restored on exposure to the light. If we reckon the specific gravity of water at 60° F. to be 1000 we find the average specific gravity of blood to be about 1055. Its general temperature is, however, from about 95° to 102°F., it is alkaline, and gives forth an odor similar to the smell of the fur or breath of the animal from which it flows.

Experiments have proven that on account of the preponderance of the red corpuscles in the male over the female, so on an average the specific gravity of the blood of the former is greater than the latter. When drawn from the body, or if it has escaped in any way from the blood vessels, and allowed to remain perfectly still, the blood undergoes some singular changes which serve to show its constituents. In about from ten to twelve minutes it assumes a jelly-like consistency without any apparent diminution in quantity or quality save that it now appears as a solid, caused by the coagulation of the fibrine together with the entanglement with it of the corpuscles, forming a clot or crassamentum. Within a short period of time this clot begins gradually to contract, thus forcing out the watery portion (serum) which retains the albumen and the saline matters, within which the clot now floats as a firmer, harder substance than before. The rapidity with which this takes place, the relative bulk of the serum and the clot, and the firmness of the latter, vary with circumstances. Spontaneous as the coagulation of the fibrine may be, yet of the above named circumstances affecting it may be mentioned the presence of an abundant supply of fresh air, for by this means the carbonic acid is more rapidly removed. The surrounding temperature greatly affects its coagulation, being greatly retarded by cold, indeed being entirely prevented by being kept at a temperature below 40° F.; in the same proportion as it is retarded by cold, so in a like proportion is it accelerated by heat until it reaches about 123° F. at and above which point it is retarded, as is also the case where death has occurred from sudden shocks as lightning. Rest has been regarded by some as necessarily essential to its coagulation; whilst, no doubt, it is a great auxilliary, yet proofs have been afforded that such is not the case, for coagulated fibrine may be readily obtained from the blood by stirring it with a small bundle of twigs. The size and firmness of the clot depends on the amount of fibrine in the blood, which in health averages about 2.3 parts in 1000. The color of the blood varies as it happens to come from the venous or arterial side of the heart. The florid scarlet arterial blood passing through the capillaries loses its oxygen becomes loaded with charbonic acid and appears in the veins of a dark purple color which it changes again for scarlet when it is sent to the lungs there to part with its carbonic acid and to absorb a fresh supply of oxygen. It seems propable that this change is owing to the effect of the oxygen on the corpuscles contracting them and altering their reflecting surfaces, carbonic acid on the other hand rendering them thinner and more flaccid.

By the process of coagulation we are furnished with the three principal constituents of the blood: 1st. the fibrine or coagulating portion, 2d. the serum, 3d. the blood corpuscles. Pure fibrine is a solid, tough, elastic substance composed of thready fibres, is whitish, inodorous and insoluble in cold water, and in healthy venous blood as before said, is about 2.3 parts in 1000. Experiments have proven that the fibrine is the only spontaneously coagulable constituent in the blood, but why this is physiologists have been unable to decide. For some length of time it has been a disputed question as to whether the coagulation of the fibrine is a process of organization or not, but more recently light thrown upon the subject by learned physiologists lead us to regard it as not being a process of organization. After the coagulation of the fibrine we have the serum remaining, it is not, however, all immediately collected, but as the clot continues to contract, more and more is pressed out, varying in different cases as to quantity as the per centage may exist in the blood and also with the rapidity with which the clot may have been formed. Its specific gravity is rather below that of the blood being only about 1028. The corpuscles are of two kinds, white and red. We are left in much doubt as to whether or not the red corpuscles are derived from those which are characterized as the white.

But making use of the best light given to us we are inclined to the opinion that the red do have their origin in the white or colorless corpuscle. The red are peculiar to vertebrate animals and to them the blood owes its characteristic color, they are circular, flattened disks varying much in size the greater proportion being in diameter about 1-3500 of an inch and from 1-8000 to 1-10000 of an inch in thickness. They are concave on both sides so that their edges are thicker than their centres and hence the dark appearance of the latter under the microscope which for sometime led observers to believe that they possessed a neucleus, but by their concavity the rays of light are reflected unequally, thus causing the dark spot in the centre. In their most perfect state they are of a uniform size and color. Being the heavier of the two are found from the laws of gravitation to collect at the bottom of the clot. When examined by the chemist the red globules of the blood consist of about 312 parts in 1000. They owe their color to a mixture of two compounds, globuline and hæmatine, the former of which on being separated crystallizes into various forms. These corpuscles perform important duties in our bodies, for possessing great powers of absorbing oxygen, they hurry away from the left side of the heart, and bear that life-giving stimulus to the tissues, takes from them, almost as readily, the carbonic acid, which, if allowed to remain would poison the whole body, carries it away and gets rid of it in the lungs where it again absorbs oxygen and again goes on its useful circuit through the body until following the laws which govern all cells and bodies composed of them they wear out, degenerate and die. The white corpuscles are greatly in the minority numbering often only one to forty or fifty of the red, they are of a light ashen appearance, circular, about 1-2800 of an inch in diameter, supposed to contain a neuclei. They are formed from masses of germinal matter thrown out from the lymphatic glands, indeed from all the ductless glands; thus appearing in the circulation as large, white, spherical, granular, neucleated cells, they go on in the process of development to form either the red blood globule or to form fibrine.

Whilst these three constituents are more generally shown by a rough analysis of the blood, there are yet, however, others which may be furnished by a chemical analysis, which may be given in such proportions as follows: Water 786. Globuline 126. Albumen 70.8. Fibrine 2.3. Saline Matmer 2. Hæmatine 5.4. These proportions are of course subject to variation, for we find the quantity of water to vary with the moisture of the atmosphere, also with the amount taken into the system. The globuline taken together with the hæmatine are difficult to distinguish at all times in their proper proportion on account of the extreme difficulty of separating them from the cell walls, and hence the chemical nature of globuline has not been accurately ascertained save that it is soluble in water. Hæmatine has a peculiar scarlet appearance which is one of its characteristics and renders it distinguishable from other animal matter, in its impure state it is soluble in water and can be washed from the blood corpuscles from which it may be obtained by precipitation. Why the peculiar red appearance should be attached to hæmatine has by some been attributed to the presence of iron, but abundant chemical experiments have been given to afford sufficient proof that such is not the case, but that rather the constant changes of color taking place during the circulation of the blood is produced by the change of form of the corpuscles themselves. The amount of fibrine varies much with the state of the health, being much reduced by fevers and also less in venous than arterial blood. To the saline matter of the blood is due its alkalinity. Fatty matter abounds in the blood to such an extent as to compose nearly if not quite all of those found in the tissues and it is to the volatile portions of these fats that the odor of the blood is due; this constituent is more abundant after a meal in which meats have been eaten. After the formation of the blood we are naturally led to inquire as to how its growth and maintenance is effected. This appears to be kept up by a repeated production of new portions from the glands and by assimilation made like it. The perfection of this assimilation is truly surprising for every tissue has its ceaseless demands to make for the portion peculiarly adapted for its sustenance which nature provides for by the Formative Power.

Being thus developed and maintained into a perfect and healthy state, surely there must be some great purposes to be accomplished which may be reckoned as three: First, to furnish to the body particles especially adapted to the nutrition and support of each portion. Secondly, to furnish oxygen to the various parts of the body. Thirdly, to bring from the various portions all debris.

---

### ARTICLE III.

### *Notes from Dental Practice.*

